# Genetic contribution to cancer risk in patients with tooth loss: a genetic association study

**DOI:** 10.1038/s41598-022-20556-2

**Published:** 2022-09-27

**Authors:** Mariana Bezamat, Scott Rothenberger, Alexandre R. Vieira

**Affiliations:** 1 Department of Oral and Craniofacial Sciences, School of Dental Medicine, University of Pittsburgh, Pittsburgh, PA USA; 2 Division of General Internal Medicine, Center for Research on Health Care Data Center, School of Medicine, University of Pittsburgh, Pittsburgh, PA USA

**Keywords:** Cancer, Genetics, Biomarkers, Diseases

## Abstract

Early-stage cancer diagnosis is critical for higher survival rates. Because early cancers can be difficult to detect, our focus is on the identification of cancer risk markers such as pleiotropic genes involved in the etiology of both craniofacial conditions and cancers. In this study we aimed to test if our previously detected association between *ERN1* rs196929 marker and oral health outcomes would be detected in individuals diagnosed with cancer as well as in a subpopulation of individuals who also had one or more teeth missing due to dental caries, periodontal disease, or periapical lesions. We genotyped a total of 1,671 subjects and selected a subset of 1,421 subjects for stratified analysis of cancer types; three hundred and twelve self-reported a diagnosis of various cancer types and 1,109 reported never receiving a diagnosis of cancer. Our results showed a statistically significant association between the rs196929 in *ERN1,* and cancer overall in both the additive and dominant models (OR = 1.37, 95% C.I. 1.06–1.79, *p* = 0.014). When we stratified the analysis for each cancer type, our results show that the rs196929 *ERN1* variant is associated with skin cancer (OR = 2.07, 95% C.I. 1.27–3.37, *p* = 0.003) and breast cancer (OR = 1.83, 95% C.I. 1.13–2.99, *p* = 0.013) in the subset of patients that had tooth loss. An additional nominal association between the rs196929 in *ERN1* and male’s reproductive system cancers (OR = 1.96, 95% C.I. 1.07–3.59, *p* = 0.028) was identified. We hope that our study helps guide future genetic studies on these cancers and this specific genetic variant as well as drive attention to the potential for oral health outcomes to serve as indicators for cancer risk. The early identification of genetic markers and/or oral conditions that indicate increased cancer risk could positively impact cancer outcomes and survival rates with timely implementation of preventive and diagnostic measures. In conclusion, our results suggest that the genetic variant in *ERN1* (rs196929) is associated with increased risk of skin and breast cancers.

## Introduction

The most common and most studied oral conditions to date include dental caries and periodontal disease, both bacteria-mediated infections^1^, and major causes of tooth loss^2,3^. Genetics has been suggested to play a role in the susceptibility to tooth loss due to both dental caries and periodontal disease^4^. Our efforts in the past years focused on the understanding of whether individuals’ genetics can influence both oral conditions such as tooth loss, and other systemic general health conditions, especially cancers^5^. Our long-term goal is to identify orofacial traits that can be markers for cancer risk and allow dental professionals to assist with cancer prevention and early cancer diagnosis by referring patients to more precise screenings.

Certain genetic pathways that regulate protein synthesis were found to influence multiple oral and systemic conditions, including metabolic disorders, type 2 diabetes and cancers^6^. Most interestingly, the endoplasmic reticulum stress (ER stress) pathway specifically, has been suggested to cause or aggravate cancers^7^, and we found it to be associated with oral health outcomes. We found that genetic variation in *ERN1* (rs196929), an ER stress variant, was associated with dental caries and periodontal disease^8^. A later study from our group showed an excess of the less common homozygote of *ERN1* rs196929 among relatives of individuals born with cleft lip and palate when they had positive family history of cancer. This pattern was similar for families that reported one type of cancer or multiple ones, or when cancer affecting females (breast or reproductive tract) or the structures of the gastro-intestinal tract were considered^9^. Moreover, a question that this previous study brought to light was whether this association could be replicated in individuals from a different geographic location and who were not born with cleft lip and palate.

In summary, our studies in the past years focused on the understanding of whether individuals’ genetics can influence both oral conditions such as tooth loss, dental caries and periodontitis and other systemic general health conditions, especially cancers^5^. We identified a knowledge gap regarding whether variation in the ER stress pathway genes play a role in cancer susceptibility the same way we found these genes to influence craniofacial conditions. With the hypothesis that the family history of cancer drove our previous results, this current study aims to test if *ERN1* rs196929 variant is overrepresented in individuals with a diagnosis of cancer using a cohort of patients going for regular dental treatment.

## Results

Our results show a statistically significant association between the rs196929 in *ERN1,* and all types of cancer combined in the genotypic (2 degrees of freedom) model (*p* = 0.0058) (Table 1). The heterozygous genotype (TC) was more frequent in individuals diagnosed with cancer as compared with matched controls (49.7% vs. 39.6%) whereas the presence of at least one copy of the allele T was more frequent in the individuals diagnosed with cancer compared to the matched controls (60% vs. 52.3%), with the results suggesting a dominant genetic model (OR = 1.37, 95% C.I. 1.06–1.79, *p* = 0.014) (Table 1).

**Table 1:** Results from the genotypic analysis of patients diagnosed with all types of cancer and the rs196929 variant (bold indicates statistically significant *p*-values under the threshold 0.05).

Chromosome	SNP	Allele1	Allele 2	TEST	Affected	Unaffected	X^2^	Degrees of freedom	*p*-value
17	rs196929	T	C	Genotypic	32/153/123	152/472/569	10.31	2	**0.0058**
		T	C	Allelic	217/399	776/1610	1.617	1	0.2035
		T	C	Dominant	185/123	624/569	5.932	1	**0.01487**
		T	C	Recessive	32/276	152/1041	1.258	1	0.262

Assuming that D is the minor allele and d is the major allele, the allelic model compares the frequencies of each allele in each group (D x d), the genotypic model is an additive two degree of freedom model that compares the frequencies of each genotype in the groups (DD x Dd x dd), the dominant model compares the two copies of the common allele frequency versus the other combinations (dd x DD + Dd), and the recessive model compares the two copies of the rare allele frequency versus the other combinations (DD x Dd + dd).

X^2^, Chi-square value.

Additionally, our results stratified by cancer type showed a statistically significant association between the rs196929 in *ERN1* and skin and breast cancers in both the logistic regression and the conditional logistic regression (Tables 2 and 3). In the logistic regression, when we stratified the analysis by each type of cancer and adjusted by age, sex, ethnicity and smoking habits we found associations between the rs196929 in *ERN1* and skin, breast and male’s reproductive system cancers (*p* < 0.05) (Table 2). When the false discovery rate was applied to adjust for multiple comparisons, only skin and breast cancer remained significantly associated with *ERN1* (Table 2). The conditional logistic regression adjusted for smoking habits confirmed the association between the rs196929 in *ERN1* and skin (OR = 2.07, 95% C.I. 1.27–3.37, *p* = 0.003) and breast cancer (OR = 1.83, 95% C.I. 1.13–2.99, *p* = 0.013) (Table 3).

**Table 2:** Logistic regression analysis of associations between the rs196929 and different types of cancer in patients with tooth loss (*p*-values below 0.05).

SNP/Allele	Cancer type	Adjustment	Odds Ratio	Lower 95% CI	Upper 95% CI	*p*-value	Number of cases	Number of controls
rs196929/T	Skin cancer	Ethnicity	1.85	1.26	2.76	**0.0019**	63	1255
		Age	1.81	1.24	2.70	**0.0025**	63	1255
		Smoking habits	1.80	1.24	2.68	**0.0027**	63	1255
		Sex	1.80	1.24	2.67	**0.0028**	63	1255
	Breast cancer	Smoking habits	1.66	1.09	2.59	**0.0193**	53	546
		Age	1.65	1.09	2.58	**0.0201**	53	546
		Sex	1.65	1.09	2.56	**0.0204**	53	546
		Ethnicity	1.65	1.09	2.57	**0.0204**	53	546
	Male’s reproductive system	Age	1.78	1.08	3.03	0.0266	37	510
		Smoking habits	1.71	1.05	2.89	0.0360	37	510
		Sex	1.68	1.04	2.84	0.0403	37	510
		Ethnicity	1.64	1.01	2.77	0.0505	37	510

Bold indicates statistically significant associations.

**Table 3:** Conditional logistic regression analysis of associations between the rs196929 and different types of cancer in patients with tooth loss (*p*-values below 0.05).

SNP/Allele	Cancer type	Adjustment	Lower 95% CI	Upper 95% CI	Odds ratio	*p*-value	Number of cases	Number of controls
rs196929/T	Skin cancer	Smoking habits	1.27	3.37	2.07	**0.003**	61	1178
	Breast cancer		1.13	2.99	1.83	**0.013**	49	514
	Male’s reproductive system		1.03	2.95	1.74	0.036	35	455

Bold indicates statistically significant associations.

From 1,421 participants included in the study, 353 were smokers and within those 71 reported a diagnosis of at least one type of cancer. From the total patients with skin cancer, 12 reported having basal cell carcinoma (with 50% carrying one copy of the variant allele and 50% carrying two copies of the variant allele), 11 reported having melanoma (with 82% carrying two copies of the variant allele and 18% carrying one copy), 5 reported having squamous cell carcinoma (with 60% carrying two copies of the variant allele and 40% carrying one copy). The remaining patients did not know or did not report the type of skin cancer in the registry. Ninety four percent of the patients reporting a diagnosis of any type of skin cancer in the study carry at least one copy of the variant allele and all the patients who reported the type of skin cancer had at least one copy of the variant allele.

We included 104 subjects in the sensitivity analysis to determine the chronology of cancer diagnosis and tooth loss. We excluded 33 subjects because they did not have enough dental treatment history available in the records since they started treatment more recently, 113 because no information on cancer diagnosis timing was available and 62 subjects because they reported a cancer diagnosis prior to 2006 and we were not able to access their oral health before that period of time. From the total of 104 individuals included, 66 had tooth loss prior to cancer diagnosis, 13 did not, 53 had tooth loss post cancer diagnosis and 30 did not have a new occurrence of tooth loss post cancer diagnosis. In approximately 24% of these subjects missing data was present and we did not consider those for analysis. Our chi-square analysis showed a significant association in this subgroup indicating that one is almost 3 times more likely to lose teeth prior to a diagnosis of cancer (OR = 2.87, 95% C.I. 1.36–6.05, *p* = 0.004).

## Discussion

Our results show a significant association between both skin and breast cancers and the rs196929 *ERN1* marker in the logistic regression and the conditional logistic regression. The positive results for this genetic association and breast cancer are consistent with our previous study^9^, and the association with skin cancer is novel. Since statistical correction for multiple comparisons might be too stringent and lead to missing potential biologically relevant results^10^, we also report nominal associations (i.e., *p* values below 0.05). We found a nominal association between this same *ERN1* marker and male’s reproductive system cancer, but this association should be interpreted with caution since there is a possibility that it is due to chance.

To date, very few studies have focused on the role of the ER stress pathway genes and skin cancer and, to the best of our knowledge, this is the first time that the rs196929 *ERN1* marker was associated with cancers. One of these few studies, however, demonstrated that, in melanoma cells, intrinsic activation of the ER stress response is caused by increased outputs of protein synthesis driven by oncogenic activation of mitogen-activated protein kinase/extracellular signal-regulated kinase (MEK/ERK) and promotes proliferation and protects against apoptosis induced by acute ER stress^11^. This might explain the association between genetic variation in the ER stress pathway and skin cancer found in this present study, given that approximately 20% of patients who reported having skin cancer had melanoma and 82% of those carry two copies of the variant allele. The rs196929 is located in an intronic region of *ERN1* and possible hypotheses on the contribution of this variant to the observed associations include that this might be a causative variant or a variant in linkage disequilibrium with another etiologically causative variant, although the exact mechanism of causation is yet to be determined.

Tooth loss has been associated with higher risk of cancers^12–16^, with factors such as behavior^17^, the oral microbiome^18^ and chronic inflammation^19^ being suggested as culprits for these associations. We have previously shown associations between genetic variation in *ERN1* and oral phenotypes, including associations with the rs196929 marker and both periapical lesions and periodontal disease^8^. In this present study we selected from our dental registry only patients who had missing teeth, however, this inclusion criterion was very broad with 85% of the patients having lost at least one tooth in the registry. Only 11% of patients were diagnosed with cancer and never had tooth loss. The inclusion of subjects that seek oral care at the dental school could be perceived as a limitation because these subjects are not representative of the general population. However, this pre-selection of patients with tooth loss might eliminate the oral health confounders of associations between cancer and genetic variation in *ERN1* such as our previously detected correlations with periodontal disease and periapical lesions*.* Another limitation was that we did not have data on patient’s diet, exercise habits, and alcohol consumption, which are variables that could impact the occurrence of cancers. Variables that we did not have data available included health behaviors, and sun exposure, which are especially important for the association with skin cancer. Furthermore, most of the subjects studied here were White, a group more susceptible to skin cancer^20^. In future studies, it would be interesting to obtain behavioral data on sun exposure and sunscreen use to correlate with oral care behavioral data and help explain the same genetic association we found for both tooth loss and skin cancer. Additionally, a future direction for these studies in the molecular level would be to determine the precise pathophysiology of these associated skin cancers for a better understanding of their etiology. Because in our study the patients self-reported their cancer diagnosis, and approximately 60% did not report the exact type of skin cancer diagnosis we were unable to precisely specify these associations. Thus, the use of self-reported data on cancer diagnosis is a limitation of this study. Although there is high accuracy in self-reporting diagnosis of cancer, especially for breast cancer^21^, we cannot discard potential issues related to recall bias of all cancer sites. Finally, because rarer cancer types have low numbers of cases in the registry, potential associations between those cancers might have not been identified due to lack of statistical power. Further, future studies that have larger sample sizes available should consider performing stratified analyses according to the reasons for tooth loss, especially for tooth loss due to caries and tooth loss due to periodontal disease which are the main contributors for tooth loss.

Strengths of our study included that we adjusted the analyses for other important variables such as smoking habits, age, sex and ethnicity. However, our results need to be confirmed with larger cohorts and different populations since the population that seeks care in our dental clinics, for the most part, lacks diversity because they are mostly White. Further, because we matched cases and controls for sex, age and ethnicity at the individual level, we performed a conditional logistic regression to confirm our results. The conditional logistic regression is more robust when individuals are matched because not only the matching process makes cases and controls similar for the variables of interest but also for the outcome status and this model corrects for this distortion^22^. The association between both skin and breast cancer and *ERN1* genetic variation remained significant in both analyses and the remaining results were also not remarkably different. We also report the number of subjects who had tooth loss pre and post cancer diagnosis considering that this oral trait could be further investigated as a risk marker for cancer in prospective and larger studies. However, we understand that cancer can be a silent disease and the diagnosis can be established several years after cancer development. This is also the case with oral conditions that lead to tooth loss such as periodontal disease and dental caries that can take many years to develop. Nevertheless, determining this chronological sequence between diseases that lead to tooth loss and cancer development is challenging and our results should be taken cautiously.

In summary, we demonstrated that *ERN1* genetic variation is associated with skin and breast cancer and potentially associated with male’s reproductive system cancers in a population that had tooth loss. We hope that our study helps inform future genetic and epidemiologic studies on cancers and the rs196929 *ERN1* marker, as well as helps drive attention to the potential for oral health indicators of general health outcomes such as cancers. The early identification of genetic markers and/or oral conditions that indicate increased cancer risk could positively impact cancer outcomes and survival rates with timely implementation of preventive and diagnostic measures.

## Methods

### Subjects

Between 2006 and 2020 a total of 6,100 individuals who sought treatment at the dental clinics were recruited to participate in the Dental Registry and DNA Repository (DRDR) project at the University of Pittsburgh, School of Dental Medicine. From those, 1,671 individuals were selected in our previous study^5^ and included in this current study: 350 because they reported a diagnosis of cancer and 1,321 that match those individuals that had cancer, based on age, sex, and ethnicity. All patient data is collected prospectively, and the medical history assessment includes a question on whether the patients were ever diagnosed with cancer as well as with other systemic conditions. These questions are asked before the dental treatments start and the answers are included as part of the medical history.

**Figure 1:**
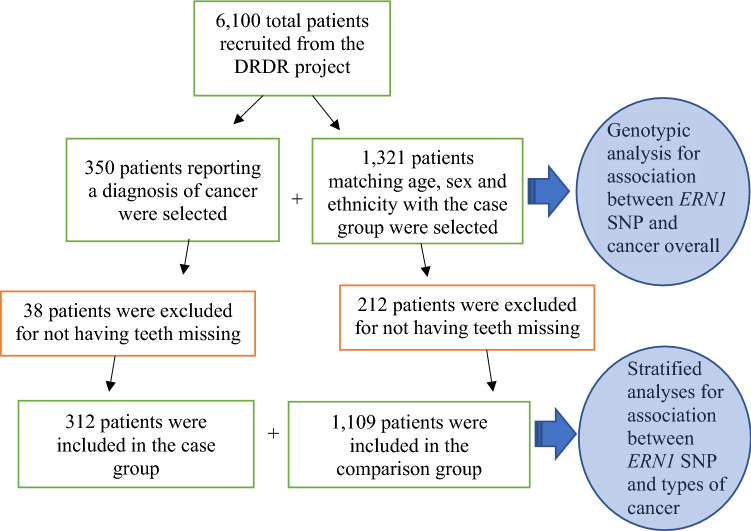
Overall study design.

We first analyzed the total sample for genetic association with the candidate gene and subsequently we performed analyses including only patients that had one or more teeth missing due to dental caries, periodontal disease, or periapical lesions. All patient evaluations and treatments are conducted at the clinics by dental students instructed by experienced professors. After excluding 250 individuals who did not have any teeth missing, a total of 1,421 subjects were included in the stratified analyses (Fig. 1). Three hundred and twelve self-reported a diagnosis of various cancer types (Table 4) and 1,109 reported never receiving a diagnosis of cancer reaching an approximate ratio of 1 case to 4 individuals for comparison. Table 5 shows the study sample demographics. Written informed consent was obtained from all participants and the project had University of Pittsburgh Institutional Review Board approval. All methods were performed in accordance with regulations, and we followed the strengthening the reporting of genetic association studies (STREGA) guidelines for this report.

**Table 4:** Types of cancers in the study sample.

Cancer type	# Cases
Skin	63
Breast	53
Male’s reproductive system	37
Female’s reproductive system	33
Head and neck	28
Colon/rectal	27
Blood	21
Urinary tract	16
Lung	11
Liver	5
Esophagus	4
Myeloma	2
Other	12
Total	312

Other = One case of osteosarcoma, 1 case of Kaposi’s sarcoma, 1 case of chest wall carcinoma, 1 case of stomach cancer, 1 case of liposarcoma, 7 unknown cases.

**Table 5:** Study sample demographics.

	Individuals with a diagnosis of cancer (n = 312)		Matched individuals without a diagnosis of cancer (1,109)	
Age in years (mean, range)	61.0	(13–90)	60.9	(18–97)
**Sex (n, %)**				
Female	155	(49.7%)	597	(53.8%)
Male	157	(50.3%)	512	(46.2%)
**Self-reported Ethnicity (n, %)**				
White	239	(76.6%)	882	(79.5%)
Black	65	(20.8%)	217	(19.6%)
Asian	1	(0.32%)	6	(0.54%)
Hispanic	3	(0.96%)	4	(0.36%)
Other	4	(1.28%)	0	(0.00%)

### Genetic polymorphism and DNA extraction

We have selected the SNP rs196929 in *ERN1* based on our previous study in which we tested 27 markers in eight genes of two cell regulatory pathways and five oral phenotypes^8^. Results showed that the SNP rs196929 in *ERN1* associated with dental caries, periodontitis, and periapical lesions. All three conditions can lead to an extreme outcome of tooth loss.

Genomic DNA was extracted from salivary samples of the 1,421 individuals using established protocols^23^. Reactions were carried out using TaqMan chemistry in volumes of 3.0 μl in an ABI PRISM Sequence Detection System 7900, software version 1.7 (Applied Biosystems, Foster City, CA, USA). Genotypes were generated blindly to clinical diagnosis status. As a measure of quality control, we used positive and negative controls as well as replicates. The variant was consistent with Hardy–Weinberg equilibrium (chi-squared *p*-value = 0.99).

### Genetic analyses

Association analyses were performed comparing genotypes and allele frequencies between the overall cancer group and the unaffected group as implemented in PLINK^24^. Models available included the allelic model that compares the frequencies of each allele in each group (D x d); assuming that D is the minor allele and d is the major allele. The genotypic model is an additive two degree of freedom model and compared the frequencies of each genotype in the groups (DD x Dd x dd), it considers that the combined effects of each allele equal the sum of their individual effects to the phenotype, the dominant model compared the two copies of the common alleles’ frequency versus the other combinations (dd x DD + Dd), and the recessive model compared the two copies of the rare alleles’ frequency versus the other combinations (DD x Dd + dd). *p*-values below 0.05 were considered statistically significant.

In order to identify the specific cancer types driving the associations, we ran both logistic regressions accounting for variables such as age, ethnicity, sex and smoking habits and conditional logistic regressions adjusting for smoking habits using the PheWAS package installed in R studio^25^. We defined smoking behavior as individuals who currently smoke every day and who have smoked at least 100 cigarettes in his or her lifetime. The diagnostic codes for types of cancer can be found at www.phewascatalog.org—the codes can be identified by either typing the correspondent ICD9 code or the phenotype of interest. This final file was then uploaded into R studio and used in the analyses. All analyses were performed using R studio version 1.4 and correction for multiple comparisons was performed using the false discovery rate to control the risk of false positive findings.

### Sensitivity analysis for timing of cancer diagnosis and tooth loss

To determine the chronology of cancer diagnosis and tooth loss, the deidentified records were assessed for the occurrence of tooth loss in the periods pre and post cancer diagnosis. We searched the Dental Registry and DNA Repository records for subjects who had a history of dental treatments done in the past years to establish this timing and perform the sensitivity analysis. We created a two-by-two table and performed a chi-square test with an alpha set to 0.05.

### Power calculations

Given N = 30 cases, N = 1250 controls, an assumed probability of exposure among control patients of 52%, and a correlation of exposure between matched individuals of 0.2, we are able to detect odds ratios as small as 3.11 with 80% power at the 5% significance level. With N = 60 cases, we can detect odds ratios as small as 2.20 with 80% power at the 5% significance level. Thus, we are adequately powered to detect odds ratios of approximately 2.20 to 3.11, which are considered medium-to-large effects per Cohen's criteria (note that OR = 1.42, 2.44, and 4.25 roughly correspond to Cohen's d = 0.20, 0.50, and 0.80, respectively).

## Data Availability

The dataset generated and/or analyzed during the current study are available from the corresponding author on reasonable request.

## References

[1] Peres MA, Daly B, Guarnizo-Herreno CC, Benzian H, Watt RG (2020). Oral diseases: A global public health challenge—Authors' reply. Lancet.

[2] Benjamin RM (2010). Oral health: The silent epidemic. Public Health Rep..

[3] Zhao X (2018). Association of periodontitis with rheumatoid arthritis and the effect of non-surgical periodontal treatment on disease activity in patients with rheumatoid arthritis. Med. Sci. Monit..

[4] Vieira AR, Hilands KM, Braun TW (2015). Saving more teeth-a case for personalized care. J. Pers. Med..

[5] Bezamat M (2020). Phenome-wide scan finds potential orofacial risk markers for cancer. Sci. Rep..

[6] Rivera Rivera A, Castillo-Pichardo L, Gerena Y, Dharmawardhane S (2016). Anti-breast cancer potential of quercetin via the Akt/AMPK/mammalian target of rapamycin (mTOR) signaling cascade. PLoS ONE.

[7] Di Conza G, Ho PC (2020). ER stress responses: An emerging modulator for innate immunity. Cells.

[8] Bezamat M (2019). Are mTOR and endoplasmic reticulum stress pathway genes associated with oral and bone diseases?. Caries Res..

[9] Assis IO (2020). IRE1 less common homozygous genotype in families with positive history of cancer and individuals born with cleft lip/palate. J. Craniofac. Surg..

[10] Vieira AR, McHenry TG, Daack-Hirsch S, Murray JC, Marazita ML (2008). Candidate gene/loci studies in cleft lip/palate and dental anomalies finds novel susceptibility genes for clefts. Genet. Med..

[11] Croft A (2014). Oncogenic activation of MEK/ERK primes melanoma cells for adaptation to endoplasmic reticulum stress. J. Invest. Dermatol..

[12] Abnet CC (2005). Tooth loss is associated with increased risk of total death and death from upper gastrointestinal cancer, heart disease, and stroke in a Chinese population-based cohort. Int. J. Epidemiol..

[13] Al-Maweri SA (2021). Association of periodontitis and tooth loss with liver cancer: A systematic review. Crit. Rev. Oncol. Hematol..

[14] Chen Y, Zhu BL, Wu CC, Lin RF, Zhang X (2020). Periodontal disease and tooth loss are associated with lung cancer risk. Biomed Res Int.

[15] Shi J (2018). Tooth loss and cancer risk: A dose-response meta analysis of prospective cohort studies. Oncotarget.

[16] Meyer MS, Joshipura K, Giovannucci E, Michaud DS (2008). A review of the relationship between tooth loss, periodontal disease, and cancer. Cancer Causes Control.

[17] Kida IA, Astrom AN, Strand GV, Masalu JR (2006). Clinical and socio-behavioral correlates of tooth loss: A study of older adults in Tanzania. BMC Oral Health.

[18] Ahn J, Chen CY, Hayes RB (2012). Oral microbiome and oral and gastrointestinal cancer risk. Cancer Causes Control.

[19] Negrini TdC, Carlos IZ, Duque C, Caiaffa KS, Arthur RA (2021). Interplay among the oral microbiome, oral cavity conditions, the host immune response, diabetes mellitus, and its associated-risk factors—An overview. Front. Oral Health.

[20] Bradford PT (2009). Skin cancer in skin of color. Dermatol. Nurs..

[21] D'Aloisio AA, Nichols HB, Hodgson ME, Deming-Halverson SL, Sandler DP (2017). Validity of self-reported breast cancer characteristics in a nationwide cohort of women with a family history of breast cancer. BMC Cancer.

[22] Kuo CL, Duan Y, Grady J (2018). Unconditional or conditional logistic regression model for age-matched case-control data?. Front. Public Health.

[23] Aidar M, Line SR (2007). A simple and cost-effective protocol for DNA isolation from buccal epithelial cells. Braz. Dent. J..

[24] Purcell S (2007). PLINK: A tool set for whole-genome association and population-based linkage analyses. Am. J. Hum. Genet..

[25] Carroll RJ, Bastarache L, Denny JC (2014). R PheWAS: Data analysis and plotting tools for phenome-wide association studies in the R environment. Bioinformatics.

